# IT Is Not for Me: Why Age Matters in Technological Innovation

**DOI:** 10.1093/geroni/igaa057.629

**Published:** 2020-12-16

**Authors:** Irene Blackberry, Clare Wilding

**Affiliations:** 1 La Trobe University, Wodonga, Australia; 2 La Trobe University, La Trobe University, Australia

## Abstract

During 2017-2019, we conducted a randomized stepped-wedge cluster trial of an online program to increase ease of access to information and support for caregivers of a person living with dementia in rural Australia. The program, called Virtual Dementia Friendly Rural Communities (Verily) Connect, was innovative; it consisted of a custom-designed website and mobile application (app) in Android and iOS, use of videoconferencing for peer support, chat forum and use of volunteers to build technological skills and confidence for online users. The program overcame barriers experienced by rural Australian caregivers, particularly geographical distance from services and other caregivers, stigma, and lack of localized supports. Although participants identified that Verily Connect program afforded several benefits, including increased flexibility in accessing support, easier access to localized dementia-related information, and more opportunities for attaining support, uptake of the program was disappointingly low. A strong theme in the program evaluation, which used the Consolidated Framework of Implementation Research, was that although the program was perceived as advantageous, adaptable, of high quality, and easy to implement, engaging participants failed because the target users, people over 65 years, eschewed involvement with online technology. Our future research plans include finding ways to offer hybrid online/face-to-face support programs specifically designed for older adults, so that older adults have equitable opportunities to access technological innovations. Older adults ought not to be disadvantaged because it is difficult to keep pace in a rapidly changing world.

